# Supporting people with type 2 diabetes mellitus through the REDE D+ social prescribing program: feasibility of a non-randomized pilot study

**DOI:** 10.3389/fpubh.2026.1822499

**Published:** 2026-06-05

**Authors:** Dulce Oliveira, Adriana Henriques, Paulo Nogueira, Andreia Costa

**Affiliations:** 1 Nursing Research Innovation and Development Center of Lisbon (CIDNUR), School of Nursing (ESEUL), Universidade de Lisboa, Lisboa, Portugal; 2 School of Nursing (ESEUL), Phd Nursing Student of Nursing School, Universidade de Lisboa, Lisboa, Portugal; 3 Department of Nursing, Escola Superior de Saúde da Cruz Vermelha Portuguesa de Lisboa, Lisbon, Portugal; 4 Instituto de Saúde Ambiental (ISAMB), Faculdade de Medicina, Universidade de Lisboa, Lisboa, Portugal; 5 Laboratório Associado TERRA, Faculdade de Medicina, Universidade de Lisboa, Lisboa, Portugal; 6 Laboratório de Biomatemática, Faculdade, de Medicina, Universidade de Lisboa, Lisboa, Portugal

**Keywords:** feasibility study, health literacy, primary care, self-care, social prescribing, T2DM

## Abstract

**Background:**

REDE D+, a social prescribing (SP) program, offers a complementary approach to traditional T2DM management by connecting people to community activities designed to support them in self-care behaviors and health literacy (HL). This study aims to assess the feasibility of a pilot study by evaluating recruitment, adherence and retention rates among people with T2DM, health professionals (nurses), community stakeholders, while exploring preliminary results in improvements on self-care behaviors and HL.

**Methods:**

This 12-week non-randomized pilot cohort study, conducted in primary and community healthcare settings in accordance with the guidelines of the Medical Research Council, involved a personalized assessment during a diabetes nursing consultation, followed by the prescription of community-based activities. Subsequently, a social prescriber nurse acts as a liaison between the two settings through structured follow-up, ensuring the continuity of the prescribed community-based activities. Primary outcomes were feasibility (recruitment, adherence, and retention rates). The secondary outcomes included anthropometric values [weight, body mass index (BMI), and abdominal perimeter], clinical values [glycated hemoglobin A1c (HbA1c)], measures of self-care, HL, diabetes knowledge, quality of life, and wellbeing. Descriptive and exploratory inferential analyses were conducted to examine preliminary patterns. Magnitude of effect was estimated via Cohen's d, allowing for a standardized assessment of improvements across the secondary outcomes.

**Results:**

The REDE D+ program revealed an 87.8% recruitment rate across the three participant groups, a 90% retention rate, and a good level of adherence to group community-based activities (94.4%). The secondary results suggest a small effect size of the SP intervention on weight, BMI, abdominal circumference, and HbA1c, and some improvements in HL and self-care behavior with a medium effect. The indicators related to physical activity and quality of life were the domains that showed the most statistically significant effects.

**Conclusions:**

The REDE D+ program has proven to be feasible, yielding positive results in terms of recruitment, retention, and adherence among people with T2DM. Preliminary secondary results suggest that has the potential to support the improvement of self-care behaviors and promote HL. However, future research is needed to evaluate its effectiveness and cost-effectiveness in large-scale studies.

## Background

Patients' conditions have been increasing in number and complexity, requiring specialized health care that poses significant challenges for individuals, health professionals, and health services (1, 2). Diabetes and its complications are also globally climbing, with 589 million people (20–79 years) worldwide requiring active and continuous diabetes self-management (3, 4). Portugal has a prevalence rate of 14.3%, one of the highest among European countries (4, 5). Diabetes self-management has a significant impact on a person's wellbeing and quality of life (QoL), as it involves acquiring new knowledge, making informed decisions, and developing different self-care behaviors (6–8). Self-care, considered an essential part of daily life for people living with diabetes, is understood as the capability of a person, family or community to maintain health and manage illness either independently or with support from health services (9). Due to its complex combination of psychosocial, behavioral and metabolic factors, multiple self-care behaviors are required, such as maintaining a balanced diet, engaging in regular physical activity, adhering to prescribed medication, self-monitoring, managing symptoms and preventing complications (10–12). Furthermore, self-efficacy and health literacy (HL) skills are essential to efficiently manage the chronic disease and navigate healthcare services and resources (13), influencing self-management behaviors that vary according to individuals' HL levels (14). HL encompasses a set of cognitive and social skills necessary for individuals to access, understand, evaluate and apply health information throughout their lives, supporting their informed decision-making (15). In the context of diabetes, HL is related to accessing and comprehending health information, following medical prescriptions, adopting care-seeking behaviors, exercising critical thinking about health information and resources received, and expressing their personal and collective needs (16). Low HL levels lead to daily challenges in managing long-term illnesses, which can affect a person's ability to plan and adapt to their lifestyle, make informed health decisions, and understand when and how to seek appropriate health services (17).

Coupled with the earlier onset of T2DM and driven by lifestyle factors, aging, and environmental influences, there is a growing need for multidisciplinary interventions tailored to each person's individual needs (18). In this context, SP intervention has established itself globally as a complex, multi-component approach (19, 20) that connects individuals to non-clinical community resources, complementing traditional healthcare and strengthening self-care capacity through integrated social responses (19, 20). It is defined as “a means for trusted individuals in clinical and community settings to identify that a person has non-medical, health-related social needs and to subsequently connect them to non- clinical supports and services within the community by co-producing a social prescription—a non-medical prescription, to improve health and well- being and to strengthen community connections”. (21) Initially, SP focused primarily on mental health and wellbeing, and has gradually expanded into other areas, such as the prevention and management of chronic diseases, with positive outcomes for health, behavior, and the economy (22, 23). Currently, SP plays an increasingly important role in addressing the needs of people with chronic conditions, such as T2DM by focusing on modifiable risk factors, such as obesity and physical inactivity, and thereby supporting meaningful lifestyle changes (24, 25). SP action also extends to the social determinants of health, minimizing their impact and creating opportunities for individuals to receive support and empowerment within their communities (26). This plays a crucial role in strengthening the motivation and continuity required for the effective self-management of chronic diseases (27). Recent studies have indicated that people at risk of developing T2DM and those already diagnosed with diabetes are the ones most frequently referred to SP interventions, reflecting the potential contribution of these interventions to behavioral change and the management of this disease (28, 29). However, research specifically examining SP in the context of T2DM remains limited. Existing studies nonetheless suggest that SP may play a significant role in preventing T2DM (28), and have shown positive results in reducing HbA1c and improving self-care and condition management (30–32).

SP has also been progressively gaining traction in Portugal as an innovative strategy. Since its initial implementation, a growing body of studies and pilot initiatives has examined its integration within the healthcare system, its perceived effectiveness, and its acceptance. The development of SP intervention has predominantly focused on promoting social wellbeing, healthy and proactive aging, with over 75% of participants considering SP to be beneficial for the healthcare system and the wellbeing of their communities (33, 34). However, while the literature describes various SP models with different delivery methods, link-worker interactions, and community contexts and activities (35–37), a significant gap remains regarding interventions specifically tailored to people with chronic metabolic disease. This study stands out for its focus on integrating self-care behaviors in T2DM and the promotion of HL as a tool for patient supported and informed decision-making. Consequently, this is the first initiative in Portugal focused on the potential of SP to support the person in better managing their T2DM, and one of the first studies to examine HL outcomes.

## REDE D+ program

The REDE D+ Program is a multi-component SP intervention, designed to support self-care behaviors and HL among people with T2DM through participation in non-clinical activities held in the community. The program's name is derived from the combination of the term REDE, equivalent to the English concept of ‘Network', the letter D, referring to diabetes, and the symbol (+), which represents the pillars guiding the program: more self-care, more health literacy, more prevention, more wellbeing and greater sustainability of healthcare. The program is based on a holistic SP model (38), in which the integration of primary healthcare with existing community resources incorporates the principles of SP through a person-centered approach, promoting the (co) development of personalized plans that address the individual's own health needs and goals. In line with the holistic SP program, it aims to encourage individuals to take a central role in managing their own care (38).

The REDE D+ Program is based on the essential self-management behaviors defined by the American Association of Diabetes Educators (AADE) (39), which have been shown to make a significant contribution to diabetes control, to empowering individuals in their decision-making, and to improving QoL, whilst reducing associated complications. These behaviors include regular physical activity, adopting a healthy diet, self-monitoring of blood glucose, adherence to the treatment regimen, self-monitoring aimed at reducing risks, as well as managing emotions and stress. By integrating community resources, the program aims to strengthen these aspects of daily self-care, offering individuals concrete and sustained opportunities for active participation in activities that contribute to the effective self-management of T2DM.

### Theoretical framework underpinning SP in the REDE D+ program

The effectiveness of behavior change interventions depends heavily on careful design and implementation (40, 41). By leveraging the *Fundamentals of Care Framework* (42, 43) to identify each person's needs and the *Behavior Change Wheel* (BCW*) Framework* (44) to provide the change and integration of new and sustained self-care behaviors. In the *Fundamentals of Care Framework*, we can see the integration of three dimensions in a person-centered approach (42, 43). At its center is the relational dimension, in which nurses establish a relationship of trust with the person, enabling them to assess, get to know them, and plan a joint intervention (42, 43). To progress to the next dimensions, identify and work with the person on their fundamental physical, psychosocial, and relationship needs. In the third dimension, he looks at the context in which care is provided, focusing on the policy (e.g., governance) and system levels (e.g., resources, culture) (42, 43). The COM-B model of the BCW framework recognizes three essential components of behavior change: (C) the person's ability to implement the change, (M) the motivation to carry out it and sustain it, and (O) the opportunity to put it into practice (44). Combined with behavior change techniques (45), this model facilitates the collaborative process of setting individual goals for each person, empowering them to develop self-efficacy and promoting positive health behaviors fundamental to T2DM self-management (46, 47). By integrating these components, the REDE D+ program aims to address the complexity of T2DM self-care behaviors, promote HL, and use the community as a valuable and strategic partner through SP intervention. Given the limited existence of SP programs at T2DM, this one stands out for providing a comprehensive, person-centered approach to empowering the person with T2DM to self-manage their condition, adopting a multimethod approach grounded in evidence-based frameworks and best practices for this study's development.

### Aims

This non-randomized cohort pilot study aims to evaluate the feasibility of a complex SP intervention (model and multi-component intervention) for supporting self-care behaviors and promoting HL in people with T2DM in the context of primary healthcare, with the following specific objectives:

- Assess the REDE D+ program's feasibility among people with T2DM, health professionals (nurses), community stakeholders.
- Assess secondary outcomes: a) anthropometric values: weight, body mass Index (BMI), abdominal circumference, and clinical values (HbA1C); b) self-care behaviors: physical activity, nutrition, wellbeing, medication management, and self-surveillance; c) HL; d) knowledge; e) QoL; and f) wellbeing.

Feasibility studies are essential in the early phases of developing and evaluating complex interventions. They help determine whether the proposed procedures, progression criteria, resources, outcomes, implementation strategies, and processes related to retention and adherence are appropriate and workable before proceeding to a full-scale evaluation (48). In this context, it is important to assess the feasibility of a non-randomized pilot study to determine whether the SP model and its multiple components are suitable and scalable in real-world primary healthcare settings.

## Methods

This non-randomized pilot cohort constitutes the fourth and final stage of the broader project ‘*Fundamental Care for the person with T2DM: Feasibility of a Complex SP Intervention'* (5078/CES/2022 ARSLVT Health Ethics Committee). Following the sequential of development of a complex intervention according to MRC, a systematic review (25), a needs assessment (49–51), and a consensus-building phase (52) to refine the intervention, were developed. This specific stage was conducted to assess the feasibility of the proposed model and a multicomponent complex SP intervention within the REDE D+ program for people with T2DM.

This pilot study is not intended to be a small-scale version of the main trial, but rather a preliminary study designed to test procedures, recruitment channels, retention, data collection processes and the operational functioning of the intervention. Pilot studies constitute a subset of feasibility studies (53), differing in their objective, which focuses on understanding and optimizing the components necessary for a future large-scale trial, rather than assessing efficacy (54). In line with these guidelines, the Medical Research Council (MRC) does not define an ideal sample size for pilot studies (53); instead, the emphasis is on ensuring that participants are representative of the target population and that the same inclusion and exclusion criteria envisaged for the future definitive trial are applied (55).

The pilot study conducted in this research aims to evaluate methods for recruiting and retaining participants, identify barriers and facilitators to implementation, and refine operational aspects of the research (56, 57). Although feasibility studies and non-randomized pilots can follow the Consolidated Standards of Reporting Trials (CONSORT) 2010 guidelines—extension for pilot and feasibility studies (57), we recognize that the non-randomized study nature imposes limitations on full compliance with these guidelines (Supplementary File 1). However, we endeavored to follow the recommendations wherever possible, which details the applicable CONSORT items that were considered.

### Study design/Setting

A feasibility non-randomized cohort pilot study was conducted between February 2024 and October 2024 and comprises three phases: the recruitment, intervention, and postintervention phase (feasibility and secondary outcomes assessment). The study was conducted at a primary care facility in the Lisbon metropolitan area and within the surrounding community. As part of this initiative, a partnership agreement was also established with a local public partner responsible for organizing community services and supporting residents and volunteers in the areas of physical activity, nutrition, and wellbeing.

### Participants/Recruitment

**Figure 1:**
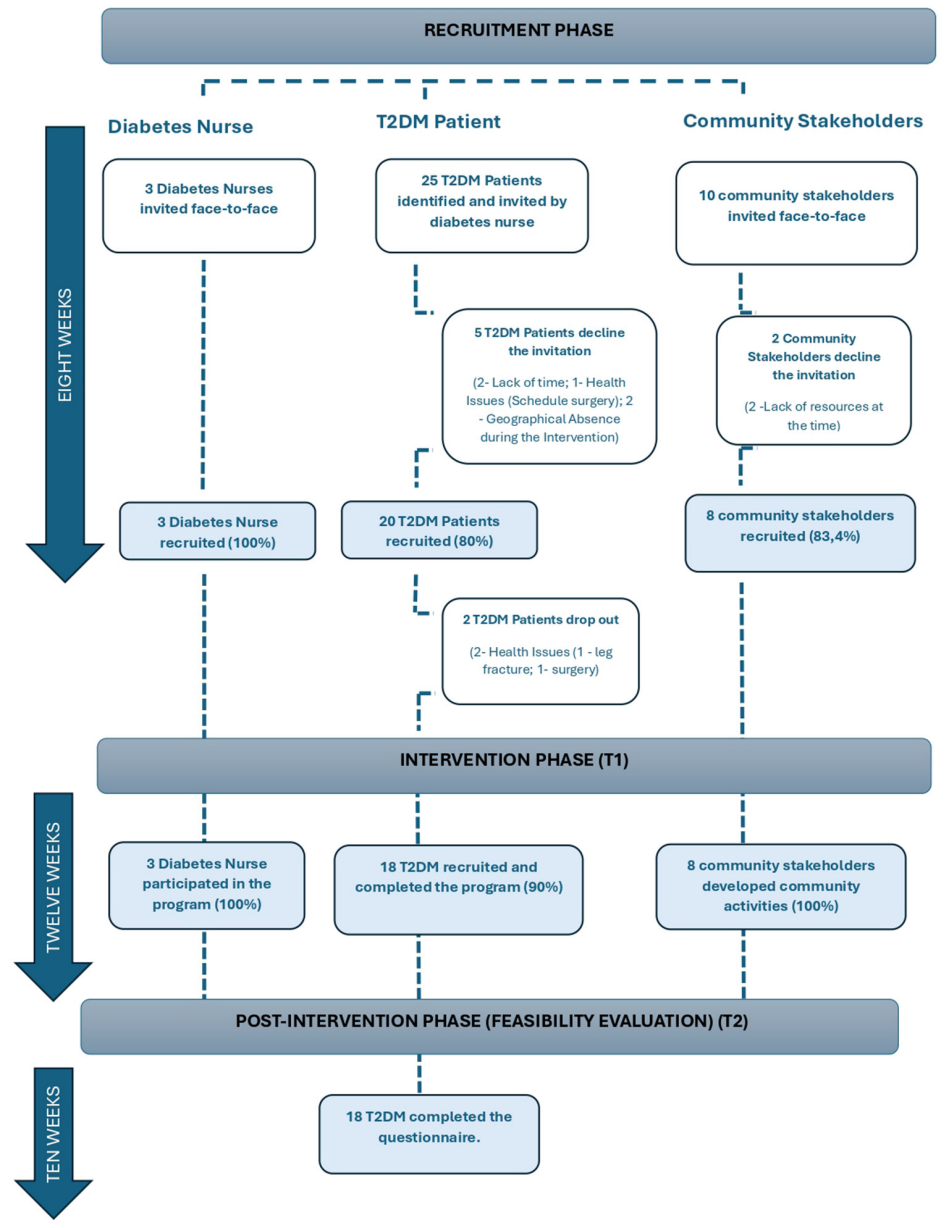
Participants flow (recruitment, intervention, and post-intervention phase).

The recruitment phase was conducted over 8 weeks, between February and March 2024. Three nurses, through a convenience sample, who participated in the previous cross-sectional study, were invited face-to-face to participate in the program. After the diabetes nurse had received prior training on the program and its resources for one hour, the diabetes nurse invited a convenience sample of 25 people with T2DM who participated in the cross-sectional study by telephone to a diabetes nursing appointment in primary healthcare. During the meeting, the nurse presented the REDE D+ program and conducted a motivational interview. A target sample of 20 participants with T2DM was recruited (Figure 1), following the recommendations for selecting the sample size for pilot studies (55, 58). After surveying existing community stakeholders who could offer a response in the areas of physical, nutrition, cultural, health, and wellbeing activities, 10 were invited to participate during a face-to-face meeting. The eight community stakeholders who accepted (Figure 1) were given a brief overview of the program and the articulation process. All participants signed an informed consent form to participate in the study.

#### Eligibility/Exclusion criteria

For the person with T2DM, anyone aged 18 years or older, with a T2DM diagnosis, followed up in a diabetes nursing appointment of the primary healthcare unit, with verbal and written knowledge of the Portuguese language, able to participate in the intervention, and mobility to go for activities in the community, were considered eligible. Any person with another type of diabetes mellitus who did not attend the diabetes nursing consultation at the primary healthcare unit, did not have the written and verbal knowledge of the Portuguese language, or had no mental or physical capacity to participate in the community activities was considered ineligible. All nurses in the diabetes nursing appointment at the primary healthcare unit who had received prior training on the REDE D+ program (recruitment, the SP intervention REDE D+ program and activities available in the communities) were eligible. Community stakeholders who are part of the community surrounding the primary health care unit, whose activities in the community meet the objectives and needs of the person with T2DM, and who have received prior training on the program, were considered eligible.

### Intervention

The Social Prescribing (SP) intervention was implemented over 12 weeks (April–June 2024) and consisted of a set of non-clinical community-based activities. The REDE D+ program followed a flowchart structured into four main stages: (1) primary healthcare nurses co-create a personalized health plan with the person; (2) referral to a social prescriber nurse within primary healthcare; (3) engagement in community activities, ensuring articulation between the healthcare and community settings; and (4) ongoing follow-up between actors to monitor progress and strengthen continuity of care.

The process begins during the diabetes nursing consultation in primary care, where the nurse, through a therapeutic relationship and a shared decision-making approach, explores the person's needs, expectations and priorities concerning T2DM self-management. The nurse makes an initial contact with the person before the program begins (T1) and performs a follow-up assessment at the end of the twelve weeks (T2). After the assessment and goal-setting phase, the nurse introduces the REDE D+ program, explaining its purpose and the potential contribution of community activities to the person's overall care plan. To support decision-making, the nurse uses a decision-support tool to prescribe community activities aligned with the person's preferences and identified needs.

**Figure 2:**
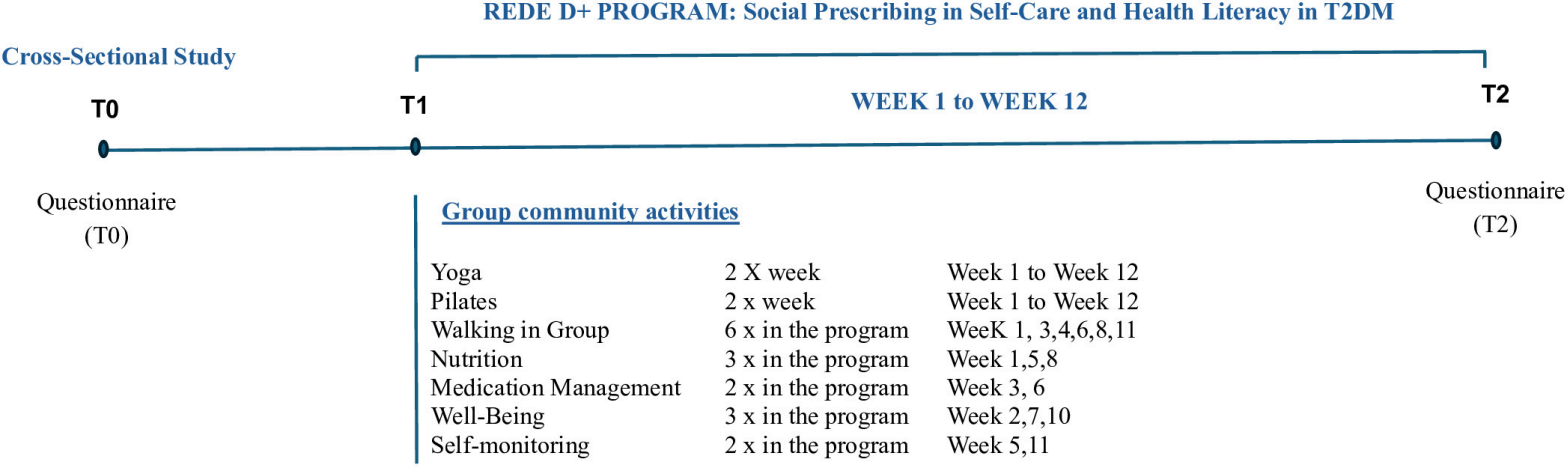
Overview of the REDE D+ program timeline, highlighting the sequence of community activities.

The decision-support tool includes the following categories of activities: Physical Activity (e.g., individual or group walking, yoga, pilates); Nutrition (e.g., healthy cooking workshops, interpretation of food labels); Wellbeing (e.g., group dialogue on emotions and stress, participation in a cultural activity); and Medication Management and Self-Monitoring (e.g., group discussions on diabetes self-management, promoting the person's active role and knowledge sharing) (Figure 2). Among all activities, walking was the only option that could be undertaken individually or in a group, depending on the participant's preference. During this initial appointment, the person received a booklet listing the available activities (the *SP passport*), where the nurse records the selected activities. The nurse informs the person that follow-up in primary healthcare will continue and that they may withdraw from the program at any time.

Following the nursing consultation, the person with T2DM establishes contact with the social prescriber, a primary care nurse and member of the research team who acts as a liaison between settings. This professional plays a key role in mediating between primary healthcare and the community. The social prescriber nurse coordinates with community stakeholders, ensuring the person's participation in the prescribed activities and providing detailed information about schedules and venues. Throughout the twelve-week program, the social prescriber nuse offers ongoing support, reviews progress and addresses any barriers or challenges that arise. Simultaneously, they maintain active communication with the community stakeholders to ensure continuity and monitor program delivery.

Community stakeholders welcome the person within the context of their respective community activities and collaborate with the social prescriber nurse to support integration and contribute to the long-term sustainability of the intervention. All community stakeholders were community partners with active roles within the community and participated on a voluntary basis. Coordination between the nurse, social prescriber nurse and community stakeholders enables a coherent, continuous and person-centered response, helping to construct a meaningful healthcare pathway aligned with the person's identified needs.

Although the initial consensus defined a broader set of activities, as well as a predetermined number and sequence of social prescriber nurse contacts, the pre-implementation review identified contextual constraints that limited their feasibility. Activities dependent on unavailable community stakeholders were removed, and both the number and the order of contacts with the social prescriber nuse were reorganized to ensure operational feasibility and alignment with real-world conditions. These modifications occurred prior to implementation and the delivered intervention reflects this final, context-adapted version while preserving its core components and intended objectives. We provide the *Template for Intervention Description and Replication* (TIDieR) Checklist (Supplementary File 2) to describe and facilitate a more comprehensive understanding of the SP intervention (59).

### Statistical analysis

Feasibility was assessed based on quantitative primary data (recruitment, adherence, and retention rates), according to the evaluation criteria previously established for the quantitative non-randomized cohort pilot study, applied to the three groups of participants. A point of ≥80% was set for each criterion to be considered met.

#### Person with T2DM

- ≥80% of the recruited participants enrolled in the REDE D+ program.
- ≥80% of participants enrolled completed both the questionnaires before (T0) and after (T2).
- ≥80% of participants remained engaged for the full 12-week duration of the REDE D+ program.
- ≥80% of participants who completed the program actively participated in the activities assigned to them.

#### Diabetes nurse

- ≥80% of the recruited diabetes nurses joined the program.
- ≥80% of diabetes nurses participated in the training session for the REDE D+ program development.
- ≥80% of diabetes nurses participated in the program development, with the first appointment (T1) after the beginning of the intervention and postintervention (T2).

#### Community stakeholders

- ≥80% of the recruited community stakeholders joined the program.
- ≥80% of community stakeholders participated in the REDE D+ program for 12 weeks.

A secondary analysis was performed by comparing quantitative data obtained from questionnaire responses collected in two moments: baseline (T0), through the cross-sectional study, and after the 12 week of the REDE D+ program (T2). The measurements collected during the initial phase served as baseline values (T0) and were compared with the post-intervention data (T2), following the implementation of the REDE D+ program. The questionnaire used can be found in Supplementary File 3 and consists of an initial section that assesses sociodemographic, anthropometric, and clinical data (HbA1C), and a second part that includes measurement scales for assessing health literacy, knowledge about diabetes, quality of life, wellbeing, and diabetes self-management.

Anthropometric variables, including height, weight, body mass index (BMI), and waist circumference, were obtained from the questionnaires and supplemented by clinical records using standardized measurement procedures. Glycemic control was assessed by extracting HbA1c values from clinical records at both time points (pre- and post-intervention). HL was assessed using the validated HLS19-Q12-PT instrument (60), which measures the individual's ability to access, understand, appraise and use health information. QoL was evaluated through the EQ-5D-5L (61), which includes five dimensions (mobility, self-care, usual activities, pain/discomfort and anxiety/depression) and a visual analog scale. Diabetes-related knowledge was measured using the Diabetes Knowledge Test (DKT) (62), covering diet, insulin use and general diabetes management. Wellbeing was assessed with the WHO-5 Wellbeing Index (63), a five-item scale evaluating mental wellbeing. Self-care behaviors were measured using the Diabetes Self-Management Questionnaire-Revised (DSMQ-R) (51), which provides an overall score and subscales for eating behavior, glucose management, medication adherence, physical activity, and cooperation with the healthcare team. Permission was sought from the authors of the scales to use them.

A descriptive and exploratory inferential statistical analysis of the quantitative data was performed via SPSS Statistics for Windows (64). The Shapiro–Wilk test was used to assess the assumption of a normal distribution, and *p* =0.05 was considered indicative of statistical significance. Given the feasibility nature of this non-randomized cohort pilot study and the absence of a control group, effect sizes were estimated through Cohen's d of observed changes and explore preliminary signals of intervention impact (small effect: d = 0.20; medium effect: d = 0.50; large effect: d = 0.80) (65) and clinical relevance was interpreted based on established standards in the literature. Preliminary data analysis can support the planning of future randomized controlled trials.

## Results

### Sample characteristics

Eighteen people with T2DM participated in the REDE D+ program for 12 weeks. As for sociodemographic and clinical characteristics, the sex distribution was predominantly female (72.2%, *n* = 13), with an average age of 68.4 years (SD ± 11.6), 61.1% (*n* = 11) had completed primary education, were retired (77, 8%, *n* = 14), and married (44.4%, *n* = 8). They were diagnosed 6 or more years (72.2%, *n* = 13), and were receiving oral therapy (88.9%, *n* = 16) (Table 1).

**Table 1:** Sociodemographic and clinical participants' characteristics.

Characteristics of participants	Frequency *(n)*	Percent (%)	Mean	*SD* (±)
Sex				
Female	13	72.2	–	–
Male	5	27.8		
Age	–	–	68.4	± 11.6
Education				
Primary education (4 years)	11	61.1	–	–
Lower secondary education (6 Years)	1	5.6	–	–
Upper secondary education (9 Years)	2	11.1	–	–
Postsecondary nontertiary education (12 Years)	3	16.7	–	–
Bachelor's degree	1	5.6	–	–
Occupation				
Self-employed	2	11.1	–	–
Retired	14	77.8	–	–
Unemployed	2	11.1	–	–
Marital status				
Married	8	44.4	–	–
Divorced	4	22.2	–	–
Widowed	6	33.3	–	–
Diabetes treatment				
Oral antidiabetic medication	16	88.9	–	–
Oral antidiabetic medication + insulin	2	11.1	–	–
Years diagnosis				
0–5 years	5	27.8	–	–
6–11 years	5	27.8	–	–
12–17 years	4	22.2	–	–
≥18 years	4	22.2	–	–

Data are n (%) or M (± SD).

### Primary outcomes

#### Feasibility (recruitment, retention, and adherence)

##### Recruitment

Through a convenience sample over 8 weeks, 25 people with T2DM who met the inclusion the inclusion criteria were deemed eligible and invited by diabetes nurses to enroll in the non-randomized pilot study. Twenty people with T2DM agreed to join the REDE D+ program, and five declined the invitation due to lack of time, health issues (scheduled surgery), or geographical absence during the intervention duration. Three diabetes nurses agreed to receive training and participated in the REDE D+ program. Ten community stakeholders were invited, but two declined due to insufficient resources at the time of the invitation. The recruitment rate of persons with T2DM was 80%, that of diabetes nurses was 100%, and that of community stakeholders was 83.4%.

##### Retention

Twenty people with T2DM that enrolled in the REDE D+ program completed the baseline questionnaire (T0) before the intervention. Eighteen patients attended the program and completed the postintervention questionnaire (T2). Two of the participants did not start the program because of health problems (leg fracture and surgery). All three diabetes nurses and ten community stakeholders participated throughout the program. The retention rate across the twelve-week intervention was 90% of persons with T2DM, diabetes nurses, was 100% and community stakeholders of 100% (Figure 1).

##### Adherence

Most of the participants took part in the proposed group activities, with an adherence rate of 94.4% (Table 2). Their adherence to each activity was assessed by signing their attendance in their SP Passport, by the community stakeholders, and follow up by the social prescriber nuse. Regarding the individual walking activities, six participants reported performing additional 30-minute walks in the community (5 days per week). Although participants were encouraged by the nurse during the consultation to keep their own records of the activity, some did not document these sessions consistently. As a result, adherence to the individual activity could not be objectively assessed. Therefore, the overall adherence rate reflects the group-based activities for which systematic and verifiable records were available. As regards follow-up appointments with the social prescriber nurse, the attendance rate was 94.4%. One patient with T2DM did not attend their appointment due to surgery.

**Table 2:** Adherence rates to group community activities.

Community activities	Number of program sessions (*n*)	Participants number (*n*)	Adherence rate (%)	Absence rate (%)	Reasons for absence
Physical activities					
Pilates	24 (2x week)	7	98.2	1.8	Medical appointment (*n* = 2)
					Health issues (*n* = 1)
Walking	6	12	98.6	1.4	Health issues (*n* = 1)
Yoga	24 (2x week)	1	100	0	–
Nutrition	3	15	100	0	–
Medication management	2	7	100	0	–
Self- monitoring	2	11	100	0	–
Wellbeing	3	11	97.0	3.0	Surgery (*n* = 1)

Data are *n* (%).

### Secondary outcomes

#### Anthropometric and clinical data

For weight, an average reduction of 1.8 kg was observed between the pre (T0) and postintervention (T2) values. The inferential analysis conducted via student's *t*-test revealed statistical significance [*t*_(18)_ = 0.911; *p* = 0.024, 95% CI (0.348; 3.374)]. Weight change showed a trivial effect (d= −0.12). BMI values reveal significant differences (*p* = 0.027) between the pre and post-intervention (T0 = 31.1 ± 4.3 kg/m^2^; T2 = 30.4 ± 3.9 kg/m^2^). Reflecting a small variation in effect size (d= −0.17). The abdominal perimeter showed an average reduction of 4 cm (T0 = 110.17 SD ± 12.1 cm; T2 = 105.70 SD ± 11.8 cm), with a statistically significant difference (*p* < 0.001). The effect analysis shows a small to medium change between T0 and T2 (d = −0.33). HbA1c levels, using the Wilcoxon test, denoted statistical significance (*p* = 0.037), with a decrease of 0.44 mg/dL and a small effect size of d = −0.21, indicating a small improvement in glycaemic control over the intervention period (Table 3).

**Table 3:** Secondary outcomes and effect sizes from baseline to post-intervention (T0 –T2).

Secondary Outcomes	Baseline (T0)		Post-intervention (T2)		Cohen's d
	*n*	*SD ±*	*n*	*SD ±*	
Weight (Kg)	82.6	± 14.6	80.8	± 14.7	−0.12
BMI (Kg/m^2^)	31.1	± 4.27	30.4	± 3.91	−0.17
Abdominal perimeter (cm)	110	± 12.1	106	± 11.8	−0.21
HbA1C (%)	6.67	± 1.58	6.39	± 1.08	−0.33
SelfCare (DSMQ-R total)	6.48	± 1.21	7.04	± 0.87	0.53
Eating behavior	6.0	± 1.73	6.91	± 1.43	0.57
Glucose management	7.37	± 1.81	7.97	± 1.75	0.34
Monitoring glucose	7.55	± 2.43	7.45	± 2.81	−0.04
Medication-tacking	7.7	± 3.27	7.84	± 1.90	0.05
Physical activity	2.38	± 1.08	6.56	± 2.66	2.06
Cooperation with healthcare team	7.71	± 1.08	8.51	± 1.62	0.58
Knowledge (DKT)	8.78	± 1.73	9.78	± 2.53	0.46
Health literacy (HLS_19_-Q12)	0.68	± 0.11	0.76	± 0.10	0.76
Health care	0.65	± 0.15	0.76	± 0.10	0.86
Disease prevention	0.66	± 0.12	0.74	± 0.14	0.61
Health promotion	0.71	± 0.11	0.78	± 0.10	0.67
Quality of life (EQ-5D-5L) VAS score	65.0	± 16	79.7	± 11.7	1.05
Wellbeing (WHO 5 index)	16.7	± 5.88	20.2	±3.73	0.71

Data are *n* and M (± SD); *d* = 0.20 (*small effect*), *d* = 0.50 (*medium effect*), *d* = 0.80 (*large effect*) according to Cohen's d (65).

##### Self-care

*A*ssessed via the DSMQ-R questionnaire revealed a medium effect in DSMQ-R total. For the Glucose Management subscale, glucose monitoring revealed a decrease in self-care-related factors (T0 = 7.55 SD ± 2.43; T2 = 7.45 SD ± 2.81), with a small effect size (d = 0.34). Improvements were also seen in the Cooperation with the healthcare team subscale, with a medium effect size. Analyzing subscales' statistical significance, only the physical activity subscale showed statistical significance (*p* < 0.001) when the Wilcoxon test was used. With an expressive large effect size (d = 2.06), the physical activity subscale showed the greatest average increase between baseline and postintervention. Followed by the eating behavior subscale (T0 = 6.057 SD ± 1.73; T2 = 6.91 SD ± 1.43), with a medium effect of d = 0.57 (Table 3).

##### Diabetes knowledge

The data from the DKT revealed improvements between the baseline and postintervention values with a small to medium effect size (d = 0.46) (Table 3). The student's *t*-test analysis revealed no statistical significance [*t*_(18)_ = −2.01; *p* = 0.061, 95% CI (−2.05; 0.05)]. However, despite this progress, the level of knowledge has remained average, indicating the need for greater attention and in-depth study in some specific areas.

##### Health literacy

Increased overall during the intervention period, with a medium effect size (d = 0.76). When the subcategories were analyzed, there was a more marked increase in the ‘Health Care' (T0 = 0.65 SD ± 0.15; T2 = 0.76 SD ± 0.10) with a large effect size intervention and ‘Disease Prevention' (T0 = 0.66 SD ± 0.12; T2 = 0.74 SD ± 0.14) with a medium effect size (Table 3). Both categories, ‘Health Care' with *p* = 0.002 and ‘Promotion Health' with *p* = 0.013, showed statistical significance according to the student's *t*-test analysis. However, the ‘Disease Prevention' category was not statistically significant (*p* = 0.057), but with a medium effect size (d = 0.67).

##### Quality of life and wellbeing

For QoL, there were improvements in the visual analog scale (VAS) scores (0–100) of people with T2DM, with a large effect size between baseline and postintervention (Table 3), with statistical significance (*p* = 0.004) according to the Wilcoxon test. Across the five scale dimensions, there was a decrease in problems related to pain/discomfort, mobility difficulties, anxiety, and depression. The improvements in wellbeing were statistically significant, with *p* = 0.004, with a medium effect size (d = 0.71).

## Discussion

This study reports on the feasibility of an SP intervention for the person with T2DM, aligned with the second phase of the MRC framework for developing complex interventions (48). This allowed the transition from the conceptual phase to the testing phase of the intervention in a real-world setting. During this phase, the study evaluated the intervention design program and its interaction in two contexts: primary healthcare and the community. Through a holistic approach, the role of the nurse in the context of diabetes and the social prescriber nuse emerges as particularly relevant, given the contributions these professionals can offer to SP interventions in chronic diseases such as T2DM. This relevance is supported by the close therapeutic relationship they establish, their specialized knowledge of T2DM, and their understanding of everyone's health challenges and needs. Existing literature highlights several key elements associated with better outcomes in SP, including adequate training of the SP, strong communication skills, and the ability to build and maintain a relationship of trust and support with the person (29, 66). Incorporating personalized goal-setting, ongoing follow-up and regular coordination between primary care and community resources through the social prescriber may contribute to favorable outcomes in self-management, adherence and metabolic control, although the extent of this influence requires further investigation through studies with larger samples.

From the outset, attention to each person's needs, barriers and self-care challenges appears to have facilitated meaningful engagement, which may partly explain the positive levels of acceptance and participation observed. In the recruitment phase, challenges arose related to the availability of patients who were committed to participating for 12 weeks. In future research, these challenges should be considered, possibly requiring greater flexibility in the program's duration and frequency or a more extended recruitment phase. However, once the REDE D+ program commenced, the recruited sample remained engaged throughout the 12-week duration, demonstrating strong adherence to the intervention's multicomponent approach. Furthermore, all previously established reliability criteria for evaluating the success of the non-randomized cohort pilot study were met, with an adherence rate exceeding 80%. One of the potential barriers that can increase the risk of dropping out or decrease retention rates for people with T2DM is related to frailty, vulnerability, and associated comorbidities (67). We can also observe that assessing adherence to individual activities, specifically walking performed independently, proved to be more challenging in this program. This finding suggests that, in future iterations of the program, it will be necessary to implement more robust methods for recording and monitoring individual activities to increase data accuracy and the reliability of adherence assessment.

Since the primary objective of this study was to assess the feasibility of implementing the REDE D+ program, the analysis of secondary outcomes should be interpreted as exploratory. These results were based on baseline questionnaire data collected before the intervention began, and there was a time interval between this baseline assessment and the start of the program. The data from the Eating Behavior subscale of the DSMQ-R, after the intervention, there were improvements in behavior with a medium effect size observed; however, there is still room for improvement. For emotional support, it is essential not to neglect the psychological wellbeing and QoL of individuals who live daily and manage chronic diseases. According to the literature, the burden of self-management of T2DM can overwhelm a person, leading to lower levels of treatment adherence and poorer health outcomes (12, 68). This program has also shown that it can have a positive influence on the person's relationship and cooperation with the team of professionals, as demonstrated by the average effect produced in the DSMQR subscale (d = 0.58). During the development of the intervention, a moderate effect size (d = 0.53) was observed for self-care behaviors, indicating an increased engagement of individuals in managing their condition. This result is in line with the principles of structured, person-centered self-care, which promote the empowerment of the person to take an active and informed part in their treatment (69). Health programs must address contributing to the support needed to help the person with T2DM achieve their health goals (68). Different techniques and resources were used as engagement strategies to facilitate the development of the SP intervention and the person's involvement. SMS messages were used as practical reminders of appointments or activities, reinforcing the importance of treatment adherence, enhancing engagement, and bringing them closer to the process (70, 71).

Although the reductions in weight, waist circumference and BMI were modest, these trends are aligned with the report by the National Academy of SP, which documents improvements in BMI observed in programs implemented in Sussex and Calderdale. (72) In addition, other studies also report weight loss associated with participation in SP interventions, reinforcing the view that this type of approach can contribute to positive changes in anthropometric indicators (30, 31, 73). These anthropometric parameters are central to the management of T2DM, as excess body weight and abdominal obesity are closely linked to insulin resistance, poor glycaemic control, and elevated cardiovascular risk (74–76). Evidence also suggests that even modest weight reductions can lead to significant improvements in metabolic outcomes and decrease the likelihood of diabetes-related complications (77, 78).

Regarding glycaemic control, a slight reduction of 4.2% (in HbA1c levels was observed, corresponding to a small effect size. Despite its modest decrease, this change holds important clinical relevance. Previous studies have demonstrated that even small reductions in HbA1c are associated with improved glycaemic control, reduced cardiovascular risk, lower incidence of complications such as chronic kidney disease, and decreased healthcare costs (79, 80).

The data reveal a substantial improvement in physical activity (d = 2.06) among the participants. These results are in line with those of previous studies demonstrating the impact of SP on promoting physical activity, reducing sedentary behavior, and improving weight and glycemic control (30, 31). However, these results should be interpreted with caution. Participants in this study reported a very low level of physical activity behaviors at baseline, which may have amplified the magnitude of the change observed after participation in the program. Furthermore, the physical activity component, which took place two to three times a week, may have contributed to the observed effect, given its greater intensity and regularity compared with other activities.

In terms of psychological wellbeing, there were improvements in the wellbeing scale with an observed medium-sized effect (d = 0.71) and large effect in QoL (d = 1.05), with a significant reduction in the anxiety/depression dimension of the quality-of-life scale after the intervention. Previous studies have shown that psychosocial problems and depression can have disruptive influences on self-care behaviors, such as physical activity and diet (81, 82). On the other hand, general wellbeing and psychological wellbeing can contribute positively to diabetes management and health outcomes (83, 84). Within the framework of the BCW, fostering psychological wellbeing is essential to strengthening the motivation (44). By addressing both reflective motivation (the conscious evaluation of goals and beliefs) and automatic motivation (emotional responses and impulses), individuals may develop the psychological readiness required to sustain long-term self-care behaviors (44, 85). Consequently, providing interventions that enhance wellbeing levels ensures that patients are more motivated to engage in self-care, leading to more enduring behaviors and a significant impact on their health status (85).

After the program, significant improvements were observed in HL and subcategories related such “health care” with a large effect size, and “disease prevention” and “health promotion” with a medium effect size, strengthening participants' ability to manage their health more proactively in the future. These results suggest an evolution in the participants' ability to manage their health and prevent complications associated with diabetes. Empowering participants to make more informed decisions not only facilitates more efficient use of available resources but also promotes the adoption of preventive practices, directly impacting the disease effects (86).

## Limitations

One key limitation of this study relates to the timing of questionnaire data collection. The baseline questionnaire was administered prior to enrolment in the REDE D+ program, rather than immediately before its implementation. This temporal gap between the baseline assessment and the start of the intervention may introduce bias and complicates the interpretation of changes observed post-intervention. In particular, this disconnect may partially account for the magnitude of effects observed for certain outcomes, such as physical activity.

Another important limitation concerns the absence of clearly defined pre- and post-intervention periods for HbA1c data collection. The use of a more structured timeline to assess HbA1c levels before and after the intervention would have strengthened the robustness of comparisons and allowed for a clearer evaluation of the program's potential impact on metabolic control. Without distinct assessment points, the interpretation of changes in HbA1c is constrained, limiting the ability to draw firm conclusions regarding the intervention's direct influence on glycaemic regulation. Overall, while the findings are broadly consistent with evidence from other studies examining the effects of SP, they should be interpreted with caution in light of these methodological limitations.

A further limitation relates to the potential challenges in implementing and transferring the REDE D+ program to multicultural contexts. The effectiveness of the intervention may be influenced by cultural differences and language barriers, particularly in communities where Portuguese is not widely spoken or understood. These factors can hinder communication, reduce engagement, and limit the impact of community-based activities, which are central to the program. Therefore, future adaptations should consider cultural and linguistic tailoring to ensure broader accessibility and effectiveness across diverse multicultural populations. Furthermore, the small sample size appropriate for a pilot study represents a key limitation in terms of statistical power and generalizability.

The limited number of participants restricts the ability to draw definitive conclusions or apply the findings broadly across the target population. Additionally, the non-randomized design limits the ability to control for potential confounding variables and increases the risk of selection bias. As this non-randomized cohort pilot study was not powered to detect statistically significant differences, the results should be interpreted as preliminary and exploratory. Although the positive trends observed are encouraging, they warrant further investigation through a randomized, controlled clinical trial with full power to rigorously evaluate the effectiveness of the intervention, confirm the results, assess the intervention's wider applicability, and its generalisability.

## Conclusions

The REDE D+ program, through a combination of tailored approaches and community-based activities, sought to ensure that people with T2DM felt supported throughout the entire SP intervention. This structured support contributed to maintaining motivation and fostering the development of skills necessary to achieve individual health goals. By adopting a flexible and adaptive structure, the REDE D+ program provided individualized responses to participants' needs, integrating two complementary contexts: primary healthcare and the community. This collaborative partnership supported a holistic, person-centered approach focused on health promotion and T2DM self-management.

The SP intervention appears to lead to improvements in self-care behaviors and significant gains across various dimensions of HL, particularly in navigating healthcare services and engaging in health promotion. This person-centered approach, integrated with community activities, helps to promote more sustained engagement in long-term diabetes self-management, as well as strengthening the individual's ability to understand, evaluate and apply health-related information. Although the effects observed at this stage of this non-randomized cohort pilot study are preliminary, they point to the positive potential that the SP intervention may have on metabolic control and the management of T2DM, improving self-care behaviors and HL, with important contributions to the QoL and wellbeing of people with T2DM. In the future, we intend to move on to the next phases of the MRC framework for the development and implementation of complex health interventions, testing the effectiveness of this SP intervention in a larger and more diverse population.

## Data Availability

The contributions presented in the study are included in the article/Supplementary material, further inquiries can be directed to the corresponding author.
