# Supplementary material for: Supporting people with type 2 diabetes mellitus through the REDE D+ social prescribing program: feasibility of a non-randomized pilot study
Source: Front Public Health. 2026 Jun 5;14:1822499. doi: 10.3389/fpubh.2026.1822499 (PMC13279591; doi:10.3389/fpubh.2026.1822499)
Supplement: Supplementary file 2 [file Table_2.docx]

| BRIEF NAME | REDE D+ Program – Social Prescribing Intervention to support Selfcare and Health Literacy in T2DM patient |
| --- | --- |
| WHY | The REDE D+ program was developed under the *Fundamental of Care Framework* and the COM-B model of Behavior  Change Wheel. Initially, it aims to identify the self-care needs of the T2DM patient and provide a behavior change by  integrating self-care behaviors developed through community activities and improving health literacy. The Behavior  Change techniques supported the development of the community's activities and resources. |
| WHAT |  |
| Materials | Nurses in the diabetes nurse consultation have an SP Manual and a decision‑support tool to support the prescription of community activities. According to the individual needs and goals T2DM patient receives an SP Kit (Backpack, booklet defined as SP Passport in T2DM, pedometer (simple step counter), and water bottle). The SP passport is a booklet that includes information about the different self-care behaviors and activities available in the community and an agenda to register the schedule of the program. The agenda is validated by community stakeholders each time the T2DM participates in the activity. During the program, the participant receives also a complementary SMS message to remind them of the activity, day and time. |
| Procedure | The 12-week program begins with an initial traditional consultation with the diabetes nurse (T1) and an invitation to take part in the REDE D+ program. During the diabetes consultation, the nurse works with the patient to identify areas of self-care behavior that can be addressed through non-clinical community activities. The nurse presents and suggests community activities that can contribute to this goal, based on the patient’s preferences. The nurse refers the patient to the social prescribing and schedules a follow-up appointment for the end of the program. After the initial meeting with the diabetes nurse, the nurse refers the patient to the prescribing nurse, who follows the person during the program. This plan can be modified or discontinued by the patient during the program, in which case the patient returns to standard care through diabetes nursing consultations.  Each person has an individualized plan of activities that can vary up to a maximum of three group activities. The community activities developed cover the areas of T2DM self-care: nutrition, medication management, physical activity, self-monitoring, and well-being. Physical activities include group walks, individual walks, Pilates, and Yoga lasting 60 minutes. Nutrition activities include cooking classes and workshops on food literacy at T2DM. In medication management, workshops on medication knowledge and literacy. In self-monitoring activities, knowledge of blood glucose values, complications, and foot care. Well-being promotion activities included a session to talk about the disease and the experience of living with T2DM, a collage session to talk about feelings and the experience of living with diabetes, a cultural activity, a visit to an exhibition, and a talk with the exhibition curator. The counseling sessions include the delivery of Behavior Change Techniques with the resources to empower and the behavior change: cards for medication registration, healthy recipe flyer provided in community activities.  Once the plan has been established, the social prescriber nurse contacts community stakeholders to inform them of the activities chosen by the individual. On the day of the activity, community stakeholders welcome the individual to the community activities and record their attendance in the SP passport. Over the course of the twelve weeks, they continue to support the individual in the various activities and maintain communication with the social prescriber nurse. |
| WHO PROVIDED |  |
|  | Nurse Diabetes (Primary Care) |
|  | Social Prescriber Nurse (Primary Care) |
|  | Community Stakeholders (Community) |
| HOW |  |
|  | The REDE D+ Program begins in the context of primary health care, with an interview and T2DM patients´ needs identification. The nurse and the person with T2DM explore what REDE D+ can offer through activities carried out in the community to improve their self-care and knowledge of the disease. Afterward, they are then referred to the social prescriber nurse, so that the latter can establish a link with stakeholders in the community. The social prescriber nurse maintains contact with the patient with T2DM throughout the program, conducting a face-to-face session at the start of the program intervention and another at the end, during which the questionnaire (T2) is administered, and making three telephone calls, or as often as necessary. The social prescriber nurse informs community stakeholders about each person’s preferences and the activities they are to undertake and stay in touch throughout the program. Community stakeholders should contact the healthcare professional responsible for the social prescribing process whenever they deem it necessary. |
| WHERE |  |
|  | Primary Healthcare and Community Context |
| WHEN and HOW MUCH |  |
|  | Physical Activity: Individual walk 30min/5 days a week; Group walks 6 x in the program with a progressive increase in intensity and duration (45min-60min); Yoga: 2 x a week 60 min; Pilates: 2 x a week 60 min. |
|  | Nutrition: 3 x in the program - 60 min |
|  | Medication Management: 2 x in the program - 60 min |
|  | Well-being: 3 x in the program - 60 min |
|  | Self-monitoring: 2 x in the program - 60 min |
| TAILORING |  |
|  | Each person defines an individual plan with the nurse diabetes to carry out in the community according to their objectives. The social prescriber, a primary care nurse, acts as a liaison between the prescription of non-clinical community-based activities by the primary care professional and the community setting. |
| MODIFICATIONS |  |
|  | Initially, the walks were planned to last between 45-60 minutes, but they were developed progressively by increasing the time (minimum of 30min (begin) to a maximum of 90 minutes (last walk)) or intensity of the route according to the community stakeholder's assessment and the individual evolution of each participant. |
| HOW WELL |  |
| Planned | Planned assessment of the participation rate in group activities. Assess the entries in the booklet (referred to as the SP passport), made by community stakeholders, regarding participants’ involvement in group activities. And assess participation in individual activities (individual walking) based on the participants’ entries in the booklet. |
| Actual | The rate of adherence and absenteeism in each group activity was analysed and can be seen in Table 2. |
