# Supplementary material for: Supporting people with type 2 diabetes mellitus through the REDE D+ social prescribing program: feasibility of a non-randomized pilot study
Source: Front Public Health. 2026 Jun 5;14:1822499. doi: 10.3389/fpubh.2026.1822499 (PMC13279591; doi:10.3389/fpubh.2026.1822499)
Supplement: Supplementary file 3 [file Table_3.docx]

**SECTION I**

ALPHANUMERIC CODE __/__

**Questionnaire - Sociodemographic and Anthropometric Characterization and Clinical Data**

Anthropometric data

Sociodemographic data

**Please select an option.**

1. AGE 7. WEIGHT 8. HEIGHT

_____ YEARS _____ KG _____METERS

2. SEX 9. IMC 10. ABD.PERIMETER

Female _____ _____ cm

Male

Clinical Data

3. EDUCATION LEVEL

Primary Education (4 years) 10. HbA1c

Lower Secondary Education (6years) _____

Upper Secondary Education (9 years) 11. Microalbuminuria

Postsecondary Education nontertiary _____

Education (12 years) 12. T2DM YEARS DIAGNOSED

Bachelor´s Degree 0 – 5 years

Master´s Degree 6 – 11 years

4. NATURALISED FROM 12- 17 years

___________________________ ≥ 18 years

5. OCCUPATION 13. DIABETES TREATMENT

Retired Diet

Self-Employed Oral Antidiabetic

Unemployed Insulin

Other _________________________ 14. TYPE OF COMPLICATION

6. MARITAL STATUS without complications

Single Diabetic Retinopathy

Married Ulcer Chronic Renal Failure

Divorced Amputation

Widowed 15. DIABETIC FOOT RISK LEVEL Low Medium High

**SECTION II**

**Diabetes Knowledge Test (DKT)**

Please circle the answer you think is correct. If you don't take insulin, answer up until question **14**. If you do take insulin, keep answering until the **end**.

**Michigan Diabetes Research and Training Center’s Revised Diabetes Knowledge Test**

**Diabetes Self-Management Questionnaire-Revised (DSMQ-R)**

|  | The following statements describe self-care activities related to your diabetes. Thinking about your self-care over the **last 8 weeks**, please specify the extent to which these statements apply to you.  Note: ‘Checking one’s glucose levels’ refers to both blood glucose testing using a meter and continuous glucose monitoring using a CGM device; please refer to your personal measurement method. | | applies to me very much | applies to me to a consider-able degree | applies to me to some degree | does not apply to me |
| --- | --- | --- | --- | --- | --- | --- |
| 1. | | I check my glucose levels with care and attention.  *Glucose checking is not required as a part of my self-care.* | 3 | 2 | 1 | 0 |
| 2. | | I structure my diet in such a way that it is easy for me to control my blood sugar properly. | 3 | 2 | 1 | 0 |
| 3. | | I regularly see the doctor (/diabetes specialist) regarding my diabetes. | 3 | 2 | 1 | 0 |
| 4. | | I take my diabetes medication (e.g. insulin, tablets) consistently and reliably.  *Diabetes medication is not required as a part of my self-care.* | 3 | 2 | 1 | 0 |
| 5. | | I occasionally eat large amounts of sweets or other foods rich in carbohydrates. | 3 | 2 | 1 | 0 |
| 6. | | I keep a diary/log of my glucose levels to improve my diabetes management.  *Glucose checking is not required as a part of my self-care.* | 3 | 2 | 1 | 0 |
| 7. | | I tend to avoid seeing the doctor (/diabetes specialist) regarding my diabetes. | 3 | 2 | 1 | 0 |
| 8. | | I am regularly physically active to improve my diabetes treatment. | 3 | 2 | 1 | 0 |
| 9. | | I follow the current dietary recommendations for people with diabetes (e.g. given to me by my doctor or diabetes specialist). | 3 | 2 | 1 | 0 |
| 10. | | I do not check my glucose levels frequently enough for achieving good blood glucose control.  *Glucose checking is not required as a part of my self-care.* | 3 | 2 | 1 | 0 |
| 11. | | I avoid physical activity although it would be good for my diabetes. | 3 | 2 | 1 | 0 |
| 12. | | I tend to forget or skip my diabetes medication (e.g. insulin, tablets).  *Diabetes medication is not required as a part of my self-care.* | 3 | 2 | 1 | 0 |
| 13. | | Sometimes I have real ‘food binges’ (not triggered by hypoglycaemia). | 3 | 2 | 1 | 0 |
| 14. | | Regarding my diabetes, I should see my doctor (/diabetes specialist) more often. | 3 | 2 | 1 | 0 |
|  | | The following statements describe self-care activities related to your diabetes. Thinking about your self-care over the **last 8 weeks**, please specify the extent to which these statements apply to you.  Note: ‘Checking one’s glucose levels’ refers to both blood glucose testing using a meter and continuous glucose monitoring using a CGM device; please refer to your personal measurement method. | applies to me very much | applies to me to a consider-able degree | applies to me to some degree | does not apply to me |
| 15. | | I am less physically active than would be good as part of my diabetes treatment. | 3 | 2 | 1 | 0 |
| 16. | | I could improve my diabetes self-care considerably. | 3 | 2 | 1 | 0 |
| 17. | | I estimate the carbohydrate content of my meals/foods (to improve my diabetes control). | 3 | 2 | 1 | 0 |
| 18. | | I eat without regard to my diabetes. | 3 | 2 | 1 | 0 |
| 19. | | I check/discuss my diabetes treatment with the doctor (/diabetes specialist) regularly. | 3 | 2 | 1 | 0 |
| 20. | | My diabetes self-care is poor. | 3 | 2 | 1 | 0 |
|  | | The following statements describe activities related to intensive insulin treatment and ***need only be answered by people using fast-acting insulin*** (i.e. people with type 1 diabetes; people with type 2 injecting insulin before meals).  If you  *don’t use insulin* OR  *use long-acting insulin only,* just leave out the following section. | applies to me very much | applies to me to a consider-able degree | applies to me to some degree | does not apply to me |
| 21. | | I check my glucose levels before each meal. | 3 | 2 | 1 | 0 |
| 22. | | I adjust my insulin doses to the carbohydrate content of my meals. | 3 | 2 | 1 | 0 |
| 23. | | I adjust the timing of my insulin injections to the start of my meals. | 3 | 2 | 1 | 0 |
| 24. | | I adjust my insulin doses according to the current glucose levels and preceding or planned activities. | 3 | 2 | 1 | 0 |
| 25. | | I correct elevated glucose levels consistently whenever necessary. | 3 | 2 | 1 | 0 |
| 26. | | I carry fast carbohydrates to enable quick treatment of low blood glucose. | 3 | 2 | 1 | 0 |
| 27. | | In case of low blood glucose, I take appropriate amounts of carbohydrates to avoid causing high blood glucose. | 3 | 2 | 1 | 0 |

© Dr Andreas Schmitt, PhD, Clin Psych, Research Institute of the Diabetes Academy Mergentheim, Diabetes Center Mergentheim, Germany. The original 16-item version of theDSMQ was developed in 2012; it was subsequently revised to this expanded version in 2015 and 2020. MAPI Research Trust®.


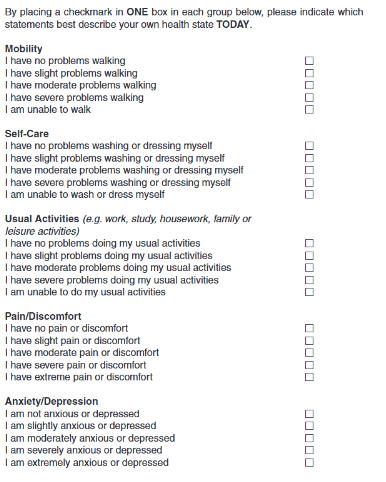


**Quality of Life - EuroQuol EQ-5D-5L**


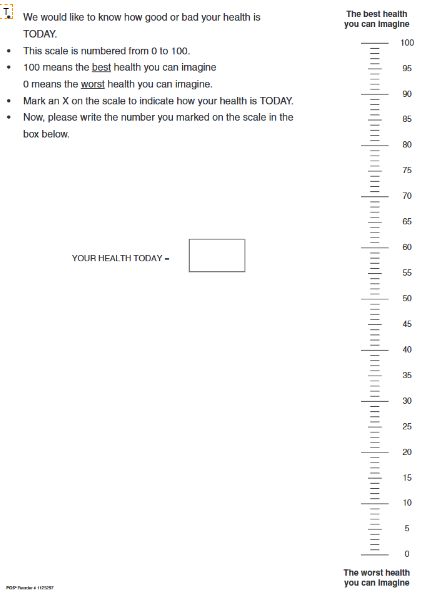


**
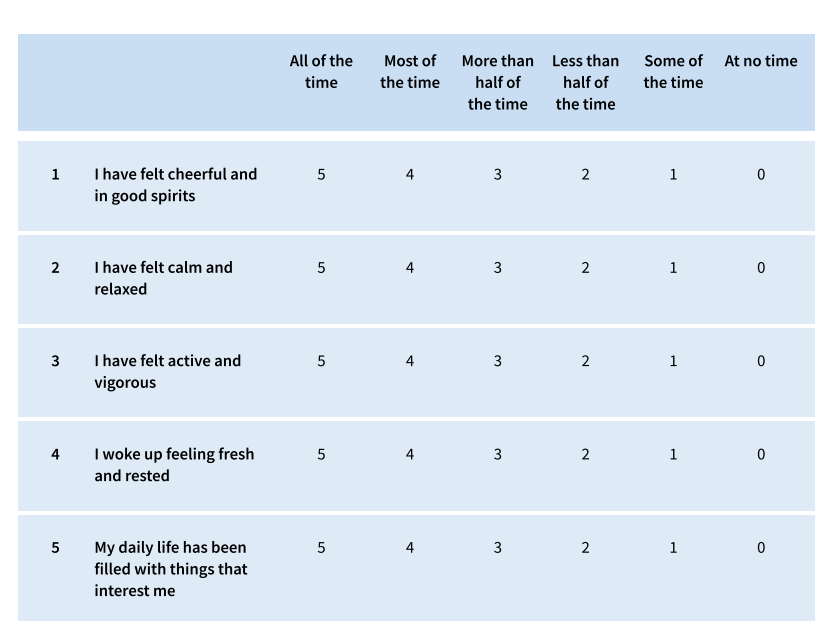
Please indicate for each of the five statements which is closest to how you have been feeling over the last two weeks. Notice that higher numbers mean better well-being.   Example. If you have felt cheerful and in good spirits more than half of the time during the last two weeks, select number three.**

**The World Health Organization-Five Well-Being Index (WHO-5)**

**Health Literacy - HLS19-Q12**

**The English version of the questionnaire HLS19-Q12 is available under request at** [**https://m-pohl.net/tools**](https://m-pohl.net/tools)**. In this research, we use the Portuguese Version under authorization.**

***Thank you for your answer!***
